# A New Code Man on Newspaper Puffs

**Published:** 1886-07

**Authors:** David Webster

**Affiliations:** New York, 266 Madison Avenue


### A NEW CODE MAN ON NEWSPAPER PUFFS.

### THE JOURNAL CRITICIZED ON ITS EDITORIAL ON PRESIDENT NUNN'S ADDRESS.

---

*To the Editors of the Atlanta Medical and Surgical Journal:*

In your editorial remarks upon "President Nunn's Address before the Medical Association of Georgia," in your issue of June, 1886, page 259, you do the so-called "new code men" an injustice, which I am sure is due to your never having seen a copy of the new code, and which I am equally sure you will cheerfully correct when put in possession of the facts. I enclose a copy of the so-called "new code." By referring to it you will find that the wording of the first paragraph is almost identical with the wording of the section of the code of the American Medical Association quoted by Dr. Nunn.

You say "such a man," *i. e.*, a physician who professes to be guided by the code of ethics, and who resorts to "the pernicious and unethical practice of causing, or allowing to be inserted in secular newspapers, paragraphs or items which have for their object the advertisement of the names of individual physicians;" "such a man can boast of neither the frankness and independence of the so-called 'new code men,' nor the honesty and fidelity to principle of those who observe the letter and the spirit of the code." Now, one would naturally infer from your remarks that such pernicious practices were approved of by the "new code men." The truth is that such methods of advertising are disapproved of equally by the adherents of both codes, and that any physician who resorts to such methods finds himself lowered in the estimation of his professional brethren.

I have yet to learn that any physician, the adherent of either code or of no code, in this city, has been so brazen as to admit that such advertisements of himself or of his operations appeared in the lay papers with his connivance or approval.

I think you will find that the code of ethics of the Medical Society of the State of New York is even more strict than the "old code," for in paragraph third it says, "It is reprehensible for physicians to give certificates attesting the efficacy of . . . . *wines, mineral waters, health resorts*, etc."—a procedure which is not forbidden by the old code.

Very respectfully yours, DAVID WEBSTER, M. D.

New York, 266 Madison Avenue, June 3, 1886.

[THE JOURNAL disclaims any intention of doing the "new code men" injustice.—EDS.]

